# AMPK activation promotes lipid droplet dispersion on detyrosinated microtubules to increase mitochondrial fatty acid oxidation

**DOI:** 10.1038/ncomms8176

**Published:** 2015-05-27

**Authors:** Albert Herms, Marta Bosch, Babu J.N. Reddy, Nicole L. Schieber, Alba Fajardo, Celia Rupérez, Andrea Fernández-Vidal, Charles Ferguson, Carles Rentero, Francesc Tebar, Carlos Enrich, Robert G. Parton, Steven P. Gross, Albert Pol

**Affiliations:** 1 Cell Compartments and Signaling Group, Institut d'Investigacions Biomèdiques August Pi i Sunyer (IDIBAPS), Barcelona 08036, Spain; 2 Department of Developmental and Cell Biology, UC Irvine, Irvine, California 92697, USA; 3 The Institute for Molecular Bioscience, The University of Queensland, Brisbane, Queensland 4072, Australia; 4 Centre for Microscopy and Microanalysis, The University of Queensland, Brisbane, Queensland 4072, Australia; 5 Departament de Biologia Cellular, Immunologia i Neurociències, Facultat de Medicina, Universitat de Barcelona, Barcelona 08036, Spain; 6 Institució Catalana de Recerca i Estudis Avançats (ICREA), Barcelona 08010, Spain

## Abstract

Lipid droplets (LDs) are intracellular organelles that provide fatty acids (FAs) to cellular processes including synthesis of membranes and production of metabolic energy. While known to move bidirectionally along microtubules (MTs), the role of LD motion and whether it facilitates interaction with other organelles are unclear. Here we show that during nutrient starvation, LDs and mitochondria relocate on detyrosinated MT from the cell centre to adopt a dispersed distribution. In the cell periphery, LD–mitochondria interactions increase and LDs efficiently supply FAs for mitochondrial beta-oxidation. This cellular adaptation requires the activation of the energy sensor AMPK, which in response to starvation simultaneously increases LD motion, reorganizes the network of detyrosinated MTs and activates mitochondria. In conclusion, we describe the existence of a specialized cellular network connecting the cellular energetic status and MT dynamics to coordinate the functioning of LDs and mitochondria during nutrient scarcity.

## Abstract (web summary)

Lipid droplets (LDs) supply fatty acids to cellular processes and move bidirectionally on microtubules. Here the authors show that nutrient starvation causes dispersal of mitochondria and LD to the periphery of the cell along detyrosinated microtubules and increases LD–mitochondria interactions in an AMPK-dependent manner.

## Graphical abstract

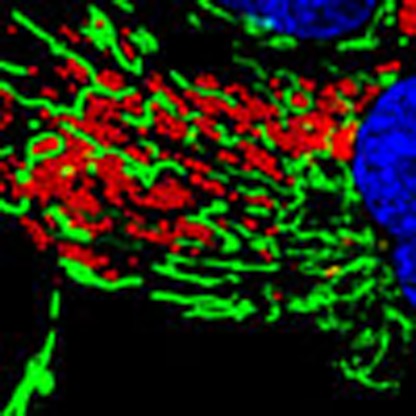

Lipid droplets (LDs) represent the main cellular lipid store and play a central role in metabolism^1^. Although adipocytes specialized for lipid storage are conserved from flies to humans, all cells store fatty acids (FAs) in LDs to balance lipid availability with metabolic and energetic demands^2^. In adipocytes, the regulation of lipolysis and the fate of the FAs stored in LDs are well characterized^3^; however, little is known about the regulation of LD metabolism in less specialized cells. Nevertheless, excessive LD accumulation in nonadipose cells is a hallmark of prevalent human diseases such as fatty liver, atherosclerosis, metabolic syndrome, heart failure and cancer cachexia^4^. Further, many pathologies and clinical manifestations arise from the central role of bioenergetics in cell biology^5^.

As lipid-storage sites, LDs have often been linked to membrane synthesis and energy metabolism^6^. The FAs stored in LDs can be used for phospholipid synthesis^7^ and for mitochondrial beta-oxidation in different cell types^8^,^9^. However, the metabolic situations where each pathway is active, and especially the mechanisms that control these different LD functions, are poorly understood. Potentially relevant is the fact that LDs sometimes show bidirectional and highly coordinated movements along microtubules (MTs)^10^. Such motion might facilitate the interaction of LDs with specific organelles and thus regulate LD different functions^11^,^12^; however, direct evidence for this is lacking.

Here we combine biochemistry, microscopy and flow cytometry analysis to determine how the cellular energetic status controls the different fates of the FAs provided by LDs, and in particular how LD location contributes to these functions. We describe a novel hierarchical cascade of events activated in response to starvation that ultimately increase FA beta-oxidation. We find a previously unidentified consortium, activated upstream by a master energy sensor (AMP-activated protein kinase, AMPK), mediated by post-translational modified MTs (detyrosinated MT), and coordinating the functioning of the two key organelles of cellular energetics (LDs and mitochondria).

## Results and Discussion

### LDs channel FAs to different metabolic fates

**Figure 1:**
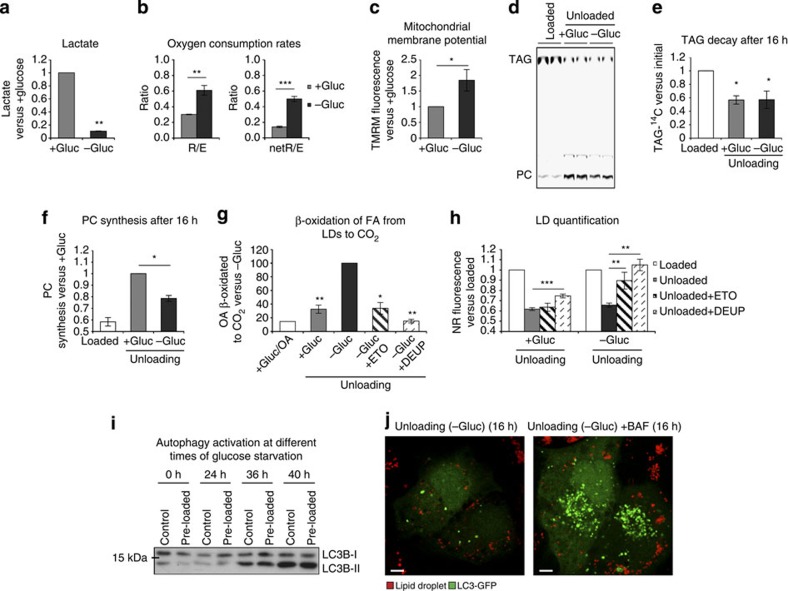
LDs channel FAs to different destinations depending on the metabolic conditions. (**a**) Lactate produced during 16 h by Vero cells in a medium either containing or lacking glucose. *n*=2. (**b**) Oxygen consumption rates in cells incubated in the presence or absence of glucose. *R/E* is routine respiration/maximal respiration capacity. Net*R/E* is the proportion of the maximal respiration capacity used to produce ATP. *n*=3. (**c**) Mitochondrial membrane potential in cells treated as in **a**. *n*=2. (**d–f**) Cells were loaded with radiolabelled FAs (Loaded) and then unloaded for 16 h in a medium either containing (+Gluc) or lacking glucose (−Gluc). A representative thin layer chromatography of the cells is shown in **d** and the quantification of triglycerides (TAG) and phosphatidylcholine (PC) in **e**,**f**. *n*=3. (**g**) Beta-oxidation in cells loaded as in **d** and additionally treated for 16 h with glucose and OA (+Gluc/OA, *n*=1), only glucose (+Gluc, *n*=4) or without glucose or FAs (−Gluc, *n*=4). Some −Gluc cells were simultaneously incubated with 100 μM ETO (*n*=3) or 500 μM diethylumbelliferyl phosphate (DEUP; *n*=3). (**h**) Quantification of the LD content in cells loaded with OA (Loaded) and additionally unloaded for 16 h in a medium either containing (+Gluc) or lacking glucose (−Gluc; *n*=14). In addition, some cells were unloaded in the presence of 100 μM ETO (*n*=5) or 500 μM DEUP (*n*=5). (**i**) Western blot analysis of LC3B in control and previously loaded cells at different times of glucose starvation. (**j**) Confocal images of GFP-LC3-expressing cells loaded with OA and unloaded for 16 h in a medium without glucose (left) or without glucose with BAF 100 nM (right), fixed and stained with Nile red (lipid droplets, red). Scale bars, 5 μm. Except for the oxygen consumption rates, data represent the mean and s.e.m. of the relative rates to an internal value of each experiment, specified in the *y* axis. *P<0.05; **P<0.01; ***P<0.001 by *t*-test.

Proliferating cells have a high FA demand for membrane synthesis^13^ and produce energy via anaerobic glycolysis by conversion of glucose to lactate^14^. In contrast, on glucose starvation, nontransformed cells remain quiescent^15^ decreasing demand for membrane synthesis and producing energy via mitochondrial aerobic metabolism and FA oxidation. Thus, in glycolytic and oxidative conditions cells should guide FAs from LDs to different fates. To analyse the utilization of the FAs stored in LDs in both metabolic conditions, we selected Vero fibroblasts. When cultured with glucose, these cells demonstrated the high lactate production and low oxygen consumption characteristics of glycolytic metabolism (Fig. 1a,b). In contrast, when glucose was removed, there is a switch from glycolytic to oxidative metabolism and cells immediately showed increased oxygen consumption, higher mitochondrial membrane potential and reduced lactate production (Fig. 1a–c). In addition, Vero cells are a good system to study this transition because they both efficiently accumulate and metabolize LDs. After incubation for 24 h with increasing doses of FAs (oleic acid, OA), cells accumulated LDs (referred to as the loading condition, Supplementary Fig. 1a,b). Further, cells metabolized the LDs after removing the supplement of FAs (unloading condition; Fig. 1h and Supplementary Fig. 1a,c). Interestingly, unloading was similar in the presence or absence of glucose, and thus these cells are able to utilize the FAs stored in LDs in both glycolytic and oxidative conditions. The consumption of LDs was identical in the presence or absence of serum and also when the unloading was performed with a delipidated serum.

Perspectives on LD utility are divergent: excessive LD storage is linked to lipotoxicity^16^; however, the ability to synthesize LDs has been suggested to be protective against nutrient starvation^17^. Thus, we examined whether overall the LDs were beneficial or detrimental for cellular survival/growth in each metabolic situation. Control cells and OA-loaded cells (loaded 24 h with OA) were unloaded for 24 or 48 h in a media either containing or lacking glucose and the number of cells was counted. In addition, cellular apoptosis was quantified after 48 or 72 h. When glucose was absent, those cells that initially contained LDs showed a significantly increased proliferation and reduced apoptosis (Supplementary Fig. 1h–k). Further, the presence of LDs also appeared useful in the presence of glucose and the cells that initially contained LDs demonstrated higher proliferation. These results suggest that for Vero cells LDs are an advantage in both glycolytic and oxidative metabolic states.

To track the fate of the FAs stored in LDs in glycolytic and oxidative conditions, we accumulated radiolabelled FAs in LDs (see Methods) and detected their localization after unloading for additional 16 h (with or without glucose). Triacylglycerol consumption (a measure of LD utilization) was confirmed using thin layer chromatography (TCL), and found that it was similar in the presence or absence of glucose (Fig. 1d,e). Although the unloading was similar, the FA destination was different: the incorporation of radiolabelled FAs into phospholipids (phosphatidylcholine) doubled during unloading in the presence of glucose (Fig. 1d,f), likely reflecting the synthesis of new membranes occurring in cells with a higher proliferation rate.

Next, we determined the extent to which FAs are channelled from LDs into mitochondria. Cells with radiolabelled FAs in LDs were again unloaded for 16 h with or without glucose. During this period, mitochondrial FA oxidation was measured by the production of radioactive CO_2_. Lipid oxidation was negligible during the loading and the unloading with glucose (Fig. 1g), reflecting a glycolytic metabolism. However, consistent with increased mitochondrial beta-oxidation, production of radioactive CO_2_ increased during the unloading in the absence of glucose (Fig. 1g). Preventing FA uptake into mitochondria with Etomoxir (ETO), a carnitine palmitoyltransferase 1 inhibitor^18^, highly reduced beta-oxidation and CO_2_ production (Fig. 1g). Interestingly, ETO completely blocked LD consumption only in the absence of glucose, supporting the model where the FAs oxidized by mitochondria are provided by LDs (Fig. 1h). Further, the FAs oxidized in mitochondria were predominantly provided by LD lipolysis because the lipase inhibitor diethylumbelliferyl phosphate^19^ completely impaired FA oxidation (Fig. 1g). In contrast, in these experimental conditions, we only detected significant activation of autophagy (accumulation of lipidated LC3, Fig.1i) after ∼36 h of removing the glucose and the FAs. Further, few authophagosomes (identified with a green fluorescent protein (GFP)-tagged LC3) were observed after 16 h of the unloading (Fig. 1j). Interestingly, the authophagosomes formed during this starvation (accumulated by using bafilomycin A1 (BAF) to prevent autophagosome degradation) were often in central regions of the cell. In contrast, most of the LDs showed a peripheral distribution (Fig. 1j), suggesting the existence of a cellular mechanism to keep away both organelles as previously proposed for mitochondria^20^. Thus, the FAs stored in LDs are channelled to different fates depending on the cellular metabolic status to phospholipids in the presence of glucose and into the mitochondria in the absence of glucose.

### Nutrient deprivation promotes LD dispersion

**Figure 2:**
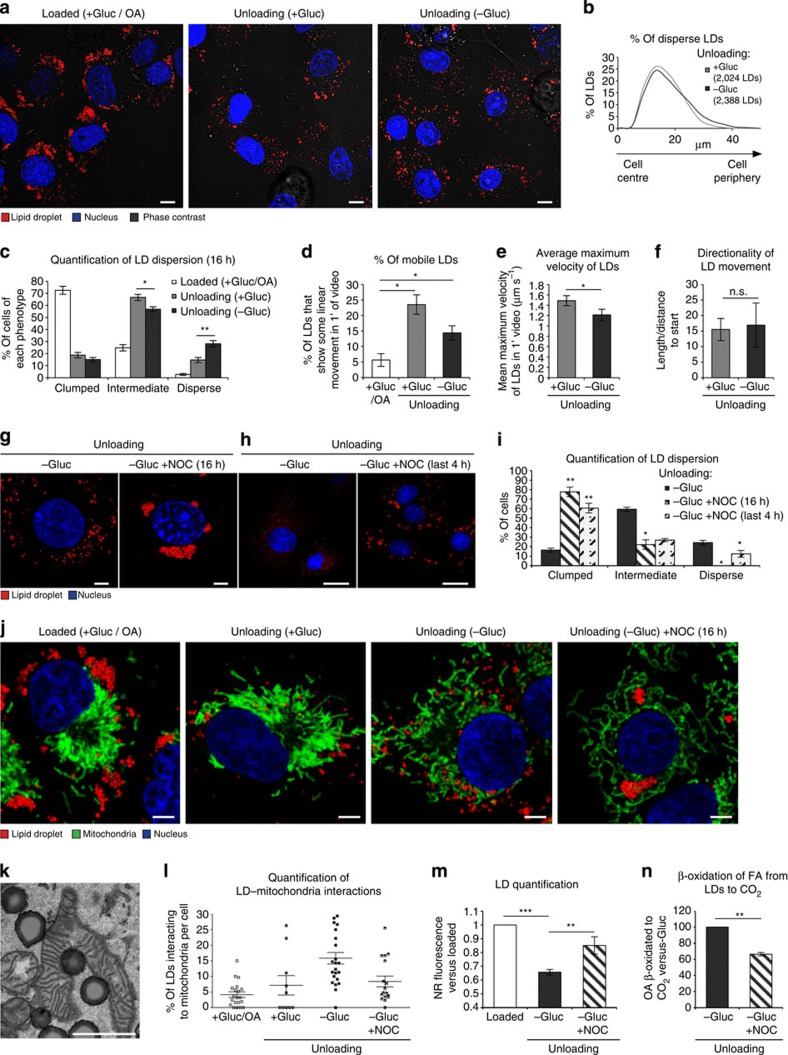
Starvation promotes MT-dependent LD dispersion and interaction with mitochondria. (**a**) Live cell confocal imaging of cells loaded with OA and additionally treated for 16 h with glucose and fatty acids (+Gluc/OA) or unloaded in a medium either containing (+Gluc) or lacking glucose (−Gluc). Cells were stained with Nile red (LD, red) and Hoechst (nucleus, blue). (**b**) Histograms representing the distance to the cell centre of the LDs of +Gluc cells (grey line, 2024 LDs) and −Gluc cells (black line, 2388 LDs). (**c**) Percentage of cells treated as in **a** and presenting three different degrees of LD dispersion: completely clumped, intermediate or completely dispersed. *n*=20, 13 and 23 independent experiments, respectively. (**d–f**) Proportion of LDs that show directional movements (**d**), average maximal LD velocity (**e**) and LD directionality (**f**) in cells treated as in **a** and analysed during 1 min (Supplementary Movie 1). *n*=3. (**g,h**) Live cell confocal imaging of cells loaded as in **a**, but unloaded for 16 h without glucose (−Gluc) or without glucose but with 15 μM Nocodazole during the 16 h of unloading (−Gluc +NOC, **g**) or during the last 4 h (−Gluc +NOC Last 4 h, **h**). Cells were stained with Nile red (LD, red) and Hoechst (nucleus, blue). (**i**) Percentage of cells treated as in (**g,h**) and presenting different degrees of LD dispersion. Statistical significance is calculated with respect the −Gluc. *n*=3. (**j**) Live cell confocal imaging of cells treated as in **a** or **g**, but stained with Deep-red mitotracker (mitochondria, green), Nile red (LDs, red) and Hoechst (nucleus, blue). (**k**,**l**) Percentage of LDs in contact with mitochondria in cells treated as in **a** or **g** and analysed using electron microscopy (34 cells and 706 LDs; **l**) and a representative image of the contacts (**k**). (**m**) LD content versus the initial LD content after loading, measured using flow cytometry of cells treated as in **g**. *n*=4. (**n**) Beta-oxidation of cells with LD loaded with radiolabelled FAs and unloaded as in **g**. *n*=3. Data are represented as mean±s.e.m. *P<0.05; **P<0.01; ***P<0.001 by *t*-test. Scale bars, 25 μm (**h**), 10 μm (**a**), 5 μm (**g,j**), 100 nm (**k**).

Because the intracellular distribution of the LDs could be important during organelle’s metabolism, we used confocal microscopy to analyse LD location before and after the removal of the FAs and glucose. After loading, cells contained numerous LDs with a relatively constant size. In most cells, LDs were clumped and formed clusters around the nucleus (Fig. 2a). In contrast, after unloading for 16 h the LDs were dispersed and, independent of the presence or absence of glucose, only few cells showed clustered LDs (Fig. 2a). However, quantification of the dispersion by classifying the proportion of cells in three different phenotypes^21^ demonstrated that the percentage of cells with completely dispersed LDs was significantly higher in the absence of glucose (Fig. 2c and Supplementary Fig. 1d for an example of each class of phenotype). The percentage of cells with highly dispersed LDs increased with longer unloading times (Supplementary Fig. 1e). During unloading with glucose, it appeared that the dispersed LDs did not disperse as far, but instead remained in more perinuclear regions. To quantify this, we developed a custom matlab code, which calculated the percentage of LDs located at least 20 μm from the cell centre. In the absence of glucose, this captured ∼32% of the cellular LDs, but for unloading with glucose, this captured ∼22% of the LDs (Fig. 2b). A very similar dispersion of LDs was observed when other cell types, such as mouse embryonic fibroblasts, COS-1 and C2C12 cells were submitted to these metabolic conditions (Supplementary Fig. 2). Moreover, confirming that LD dispersion is a starvation-dependent process, the inhibition of glycolysis with 2-deoxyglucose promoted LD dispersion even in the presence of glucose and FAs in the media (Supplementary Fig.1f,g).

In order to characterize LD dispersion, we analysed LDs using video microscopy and measured LD dynamics. After loading, when FAs were present in the medium, the resulting LDs were relatively static. Only an average ∼5% of the LD in each cell showed some fast directional movements during 1 min (Fig. 2d and Supplementary Movie 1). In contrast, in cells unloaded for an additional 16 h, the number of mobile LDs per cell increased 3–4X, and ∼15–20% of the LDs changed position during a very short period of 1 min (Fig. 2d and Supplementary Movie 1). Although the directionality of the movement was similar, the number and the velocity of mobile LDs were slightly higher in the presence of glucose (Fig. 2d–f). Thus, depletion of FA promotes increased LD mobility that likely contributes to LD dispersion.

LDs display regulated motion driven along MTs in different cell types; however, the underlying utility or mechanism controlling such motion is poorly understood^10^. We confirmed contribution of MT to LD dispersion by treating cells with Nocodazole, a MT-depolymerizing agent. Nocodozale completely inhibited the LD dispersion promoted by the unloading (Fig. 2g,i). Importantly, Nocodazole treatment as short as 4 h was sufficient to completely reverse the dispersion caused by the 16 h of nutrient deprivation (Fig. 2h,i). Video microscopy using a fluorescent tubulin confirmed directly that during unloading, LDs move on MTs (Supplementary Movie 2). Therefore, MTs are required to both promote and maintain LD dispersion.

### Nutrient starvation promotes LD–mitochondria interaction

Since in the absence of glucose the LDs provide FAs to mitochondria, we next compared the intracellular distribution of both organelles. After FAs and glucose removal, the images confirmed the previously described mitochondrial dispersion^20^, and importantly showed that the majority of dispersed LDs were apparently in contact with mitochondria (Fig. 2j). For better resolution, we quantified the number of LD–mitochondria contacts using electron microscopy (Fig. 2k and Supplementary Fig.3). We first looked for interactions LD–mitochondria in loaded cells and in cells unloaded with glucose. In each, direct LD–mitochondria contacts were observed only in ∼50% of the cells. In these cells, on average 4.1 or 7.1% of the LDs were, respectively, interacting with mitochondria (Fig. 2l). In contrast, we observed LD–mitochondria contacts in 100% of the cells unloaded for 16 h in the absence of glucose. Now, an average of 16.5% of the LDs had at least one, and often two, contacts with mitochondria (Fig. 2l), reflecting a 3.9-fold increase in contacts when compared with loaded cells.

Some LD–mitochondria interactions were apparently strong and often the mitochondrial membranes were deformed around the LDs (Fig. 2k and Supplementary Fig. 3f–i). Importantly, in addition to inhibiting LD dispersion, Nocodazole significantly reduced the number of LD–mitochondrial contacts to 7.3% of the LDs (Fig. 2j,l), LD consumption (Fig. 2m) and beta-oxidation of the FAs stored in LD (Fig. 2n). Finally, the dynamics of the LD–mitochondria interaction were analysed using *in vivo* confocal microscopy. Using this approach, we detect multiple types of interactions. We observed both kiss-and-run and relatively stable contacts (Supplementary Fig. 3a–d and Supplementary Movie 3). In addition, some LDs were stretching, and in some cases breaking, mitochondria (Supplementary Fig. 2a,b and Supplementary Movie 3), clearly suggesting the existence of a mechanical force driving the interaction. Thus, after FA and glucose removal, the MTs contribute to dispersion to increase the number of LD–mitochondria contacts, the oxidation of FAs and the consumption of LDs.

### A subset of MTs contributes to LD dispersion

**Figure 3:**
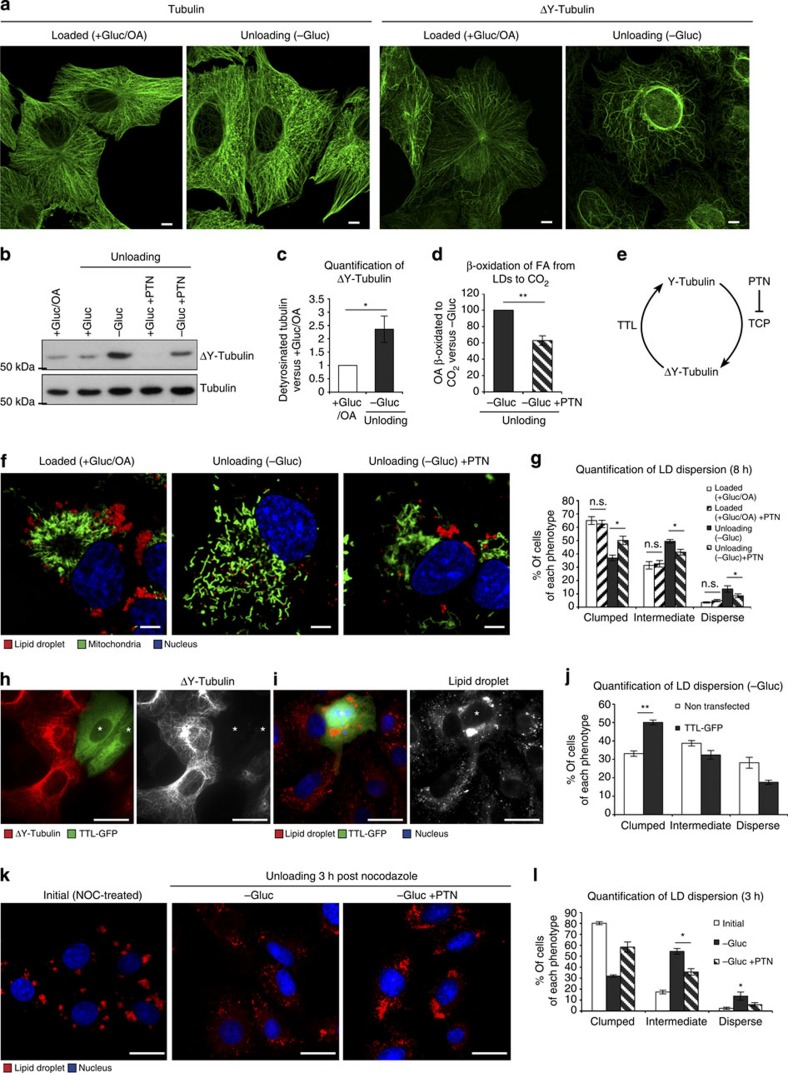
MT detyrosination is required for LD dispersion and LD–mitochondrial contacts. (**a**) Confocal images of cells loaded with OA and additionally treated for 16 h with glucose and FAs (+Gluc/OA) or without glucose (−Gluc). Cells were fixed and labelled with an antitubulin (left panels) or antidetyrosinated tubulin antibodies (right panels). (**b**) Determination of detyrosinated tubulin and tubulin levels using western blot analysis. Cells loaded as in **a** were then treated 8 h with glucose and FAs (+Gluc/OA) or unloaded in a medium either containing (+Gluc) or lacking glucose (−Gluc). Some cells were unloaded in the presence of 15 μM PTN. (**c**) Quantification of the ratio of detyrosinated tubulin versus total tubulin, normalized with respect to the +Gluc/OA condition, in cells analysed as in **b**. *n*=8. (**d**) Beta-oxidation in cells loaded with radiolabelled FAs and unloaded for 16 h in a medium without glucose (−Gluc) or without glucose but with 15 μM Parthenolide (−Gluc +PTN; versus −Gluc). *n*=3. (**e**) Scheme of tubulin detyrosination enzymes. (**f**) Live cell confocal imaging of cells treated as in **b** but stained with Deep-red mitotracker (mitochondria, green), Nile red (LD, red) and Hoechst (nucleus, blue). (**g**) Percentage of cells treated as in **b** and presenting different degrees of LD dispersion. *n*=5. (**h**,**i**) GFP-TTL-transfected cells were loaded with OA and treated for 16 h without glucose. Cells were fixed and labelled with antidetyrosinated tubulin antibody (**j**) or stained with Nile red (red) and DAPI (nucleus, blue) (**k**). (**j**) Percentage of cells treated as in **k** and presenting different degrees of LD dispersion. *n*=2. (**k**,**l**) Cells were loaded with OA and treated for 4 h without glucose in the presence of 15 μM Nocodazole (initial). Next, Nocodazole was washed out and cells were incubated for 3 h (post nocodazole) without glucose (−Gluc) or without glucose and with 25 μM Parthenolide (−Gluc +PTN). Cells were fixed and stained with Nile red (red) and DAPI (nucleus, blue). The images are showed in **h** and quantification of LD dispersion in **i**. *n*=2. Data are represented as mean±s.e.m. *P<0.05 and **P<0.01 by *t*-test. Scale bars, 7.5 μm (**a**,**f**) and 25 μm (**h**,**i**,**k**).

Since MT-based LD transport contributes to upregulation of beta-oxidation, we next investigated whether starvation might somehow regulate the MT network. We analysed overall tubulin organization with immunofluorescence after nutrient starvation. During the unloading in the absence of glucose, the MT network remained organized radially, although some dot-like structures were formed (Fig. 3a). Some post-translational MT modifications such as acetylation (Ac-MT) participate in the interaction between mitochondria and the endoplasmic reticulum^22^, and this modification is reported to increase during starvation^23^. We confirmed an increase in Ac-MTs after glucose removal; however, reduction of Ac-MTs by knockdown of alpha-tubulin acetyltransferase had little effect, suggesting that Ac-MTs are not required for LD dispersion (Supplementary Fig. 4). We next investigated the role of detyrosinated MTs (ΔY-MTs). In mammals, the C-terminal tyrosine of alpha-tubulin is subject to cyclic removal by a tubulin-carboxypeptidase (TCP) exposing a glutamic acid, potentially followed by re-addition of the tyrosine by a tubulin–tyrosine ligase (TTL)^24^(Fig. 3e). Thus, we tested whether ΔY-MTs changed and found that in Vero cells the unloading without glucose caused an increase in detyrosinated tubulin (Fig. 3b,c). The immunocytochemistry images demonstrated dramatic changes in the organization of the ΔY-MTs depending on nutrient availability. In loaded cells, the ΔY-MT network was radially organized and centred on the MT-organizing centre (Fig. 3a). In contrast, in cells unloaded without glucose the ΔY-MT network organized in a noncentrosomal array (right panel in Fig. 3a). A similar organization of ΔY-MT, related with higher MT stability, was previously only reported in epithelial cells^25^.

To test whether the ΔY-MT network contributes to LD dispersion, cells were unloaded in the presence of Parthenolide (PTN), an inhibitor of TCP (the enzyme that produces ΔY-tubulin, Fig. 3e), which efficiently reduced levels of ΔY-tubulin (Fig. 3b). Quite strikingly, during unloading in the absence of glucose, PTN highly reduced LD dispersion (Fig. 3f,g). In contrast, PTN had little effect on dispersion when cells were unloaded with glucose (Supplementary Fig. 5d). Functionally, identical to Nocodazole (Fig. 2n), and consistent with the clumped LD distribution, PTN reduced the beta-oxidation of FAs from LDs after the unloading without glucose (Fig. 3d). Importantly, PTN also inhibited LD dispersion and FA oxidation in more oxidative cells such as C2C12 myoblasts (Supplementary Fig. 2c,d). Although we focused our work on LD distribution, PTN also reduced the mitochondrial redistribution observed after FA removal in the absence of glucose (Fig. 3f), pointing to a role of ΔY-MTs in mitochondrial distribution as well. To further demonstrate that the reduction of the ΔY-tubulin levels reduced LD dispersion, a GFP-tagged TTL (Fig.3e) was transfected in cells. Indeed, expression of TTL drastically reduced the network of ΔY-MTs formed in response to the unloading without glucose (Fig. 3h) and significantly inhibited LD dispersion (Fig. 3i,j).

Interestingly, treatment with PTN for 1 h in cells previously unloaded without glucose for 16 h also promoted a clustered LD distribution (Supplementary Fig. 5e), suggesting that ΔY-MTs are required to maintain the LDs in the cell periphery. To test whether ΔY-MTs also mediate the dispersion process, loaded cells were treated with Nocodazole for 4 h to initially depolymerize MTs. Next, Nocodazole was washed out, to allow MT’s regrowth, in a media without glucose but in the absence or presence of PTN, to specifically prevent ΔY-MT’s re-formation. In both cases, MTs were re-polymerized after 3 h of Nocodazole removal (Supplementary Fig. 5a). However, when compared with the cells recovered without PTN, in which the starvation-promoted noncentrosomal ΔY-MTs were reformed after 90 min, the presence of PTN clearly inhibited the regrowth of ΔY-MTs and, importantly, the dispersion of LDs occurring during this short period of starvation (Fig. 3k,l and Supplementary Fig. 5a–c and Supplementary Movie 4). Thus, ΔY-MTs are required to promote and maintain LD dispersion and to increase LD consumption during nutrient deprivation.

**Figure 4:**
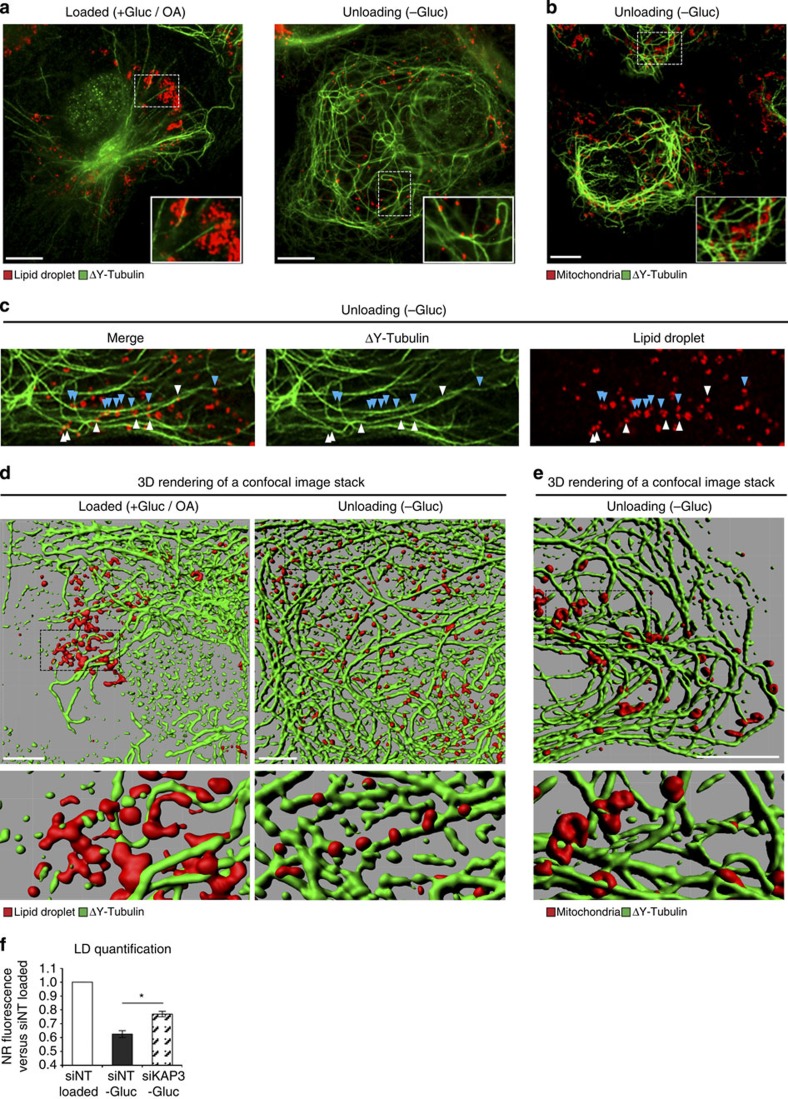
LD and mitochondria interact with detyrosinated microtubules in starvation. (**a**) Images of cells loaded with OA and additionally treated for 16 h with glucose and FAs (+Gluc/OA) or unloaded without glucose (−Gluc). Cells were fixed and labelled with an antidetyrosinated tubulin (green) and anti-ACSL3 (LDs, red). (**b**) Confocal image of cells loaded with OA and unloaded for 16 h without glucose. Cells were fixed and labelled with an antidetyrosinated tubulin (green) and anti-ATPsynthase (mitochondria, red). (**c**) Detail of a confocal image of an unloaded cell treated as in **a** (right). Blue and white arrows indicate the LDs attached to two different detyrosinated microtubules. (**d**,**e**) 3D-rendering (Imaris, Bitplane) of a confocal image stack of cells treated as in **a**,**b**, respectively. (**f**) LD content measured using flow cytometry in cells transfected with an siRNA nontargetting (siNT) or siRNA against KAP3, loaded with OA (loaded) and treated for 16 h without glucose. *n*=4. Scale bars, 10 μm (**a**,**b**,**e**), 5 μm (**d**, left), 4 μm (**d**, right). *P<0.05 by *t*-test.

Thus, we next analysed whether LDs disperse on ΔY-MTs. Loaded and unloaded cells were labelled with antibodies against ΔY-tubulin and ACSL3, a LD marker^26^. For loaded cells, as expected, the LDs accumulated in perinuclear regions and the network of ΔY-MT was radially organized (Fig. 4a). Although some contacts between droplets and tubules were evident, most of the LDs did not show an apparent interaction with ΔY-MTs. In striking contrast, in response to the unloading without glucose all the LDs contacted the noncentrosomal ΔY-MT network (Fig. 4a). Rows of LDs that were detected aligned along the ΔY-MTs (high-magnification panel in Fig. 4c). The LDs were preferentially associated with the peripheral tubules but they were also associated with the rounded ΔY-MTs of the cell centre. Interestingly, when the distribution of mitochondria was analysed in unloaded cells—with an ATP synthase antibody—these organelles also distributed preferentially along the ΔY-MT (Fig. 4b). In contrast to LDs, mitochondria were often surrounded by ΔY-MTs (insert Fig. 4b). Owing to the complex three-dimensional (3D) organization of the MT network, the interaction between these organelles and the MTs was further analysed rendering confocal microscopy sections by means of the Imaris software. The 3D reconstruction confirmed the results obtained using conventional microscopy. Whereas few LDs were in contact with the ΔY-MTs in loaded cells, the totality of the LDs of unloaded cells contacted the network of ΔY-MTs (Fig. 4d). Similarly, the majority of the mitochondria appeared anchored to ΔY-MTs (Fig. 4e). Thus, these results demonstrate that during unloading without glucose the LDs and mitochondria are located along the same ΔY-MT tracks, likely increasing proximity and interaction between the organelles.

Next, we looked for the motors that moved the LDs on the ΔY-MT tracks. The short interfering RNA (siRNA)-mediated downregulation of kinesin-1 did not produce any effect on LD consumption. However, after downregulation of kinesin-2 with a siRNA of KAP3 (ref. 27; to 50% of the original levels, as measured using real-time PCR), we observed a modest but significant reduction in the consumption of LD during the unloading without glucose (Fig. 4f), suggesting that kinesin-2 is the motor protein that mediates, at least partially, LD consumption.

### AMPK activation promotes LD dispersion and consumption

**Figure 5:**
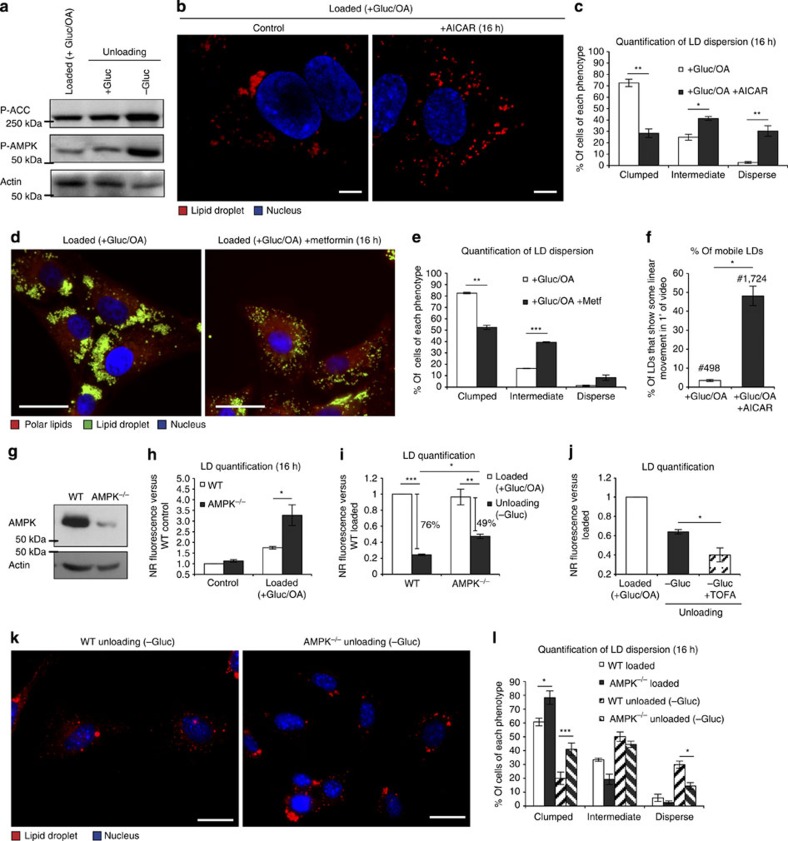
AMPK promotes LD mobility, dispersion and consumption. (**a**) Western blot analysis of phosphorylated ACC (upper panel), phosphorylated AMPK (middle panel) and total actin (lower panel). Cells were loaded with OA and treated for 16 h with glucose and OA or unloaded in a medium with or without glucose. (**b**,**c**) Live cell imaging (**b**) and quantification of LD dispersion (**c**) of cells loaded and treated for 16 h with glucose and FAs in the absence (Control) or presence of 2.5 mM AICAR. Cells were stained with Nile red (LD, red) and Hoechst (nucleus, blue). *n*=4. (**d**,**e**) Images (**d**) and quantification of LD dispersion (**e**) of loaded cells treated for 16 h in the absence (+Gluc/OA) or presence of Metformin 50 mM (+Gluc/OA +Metformin). Cells were fixed and stained with Nile red (red for polar lipids and green for neutral lipids) and DAPI (nucleus, blue). *n*=2. (**f**) Proportion of LDs that show directional movements during 1 min in cells treated as in **b** (Supplementary Movie 3). The number of LDs analysed are specified in the corresponding bar. # indicates the number of LDs quantified. (**g**) AMPK and actin levels determined with western blot analysis in WT and AMPK^−/−^ MEF cells. (**h**) LD content measured with flow cytometry in MEF WT and AMPK^−/−^ cells treated for 24 h in control medium (Control) or with 175 μg ml^−1^ OA (Loaded). *n*=3. (**i**) LD content measured in WT and AMPK^−/−^ cells loaded with 400 and 200 μg ml^−1^ OA, respectively, and unloaded for 16 h without glucose (−Gluc). *n*=3. (**j**) LD content measured in cells loaded and unloaded for 16 h without glucose; with or without TOFA 30 μM. *n*=3. (**k**,**l**) Images (k) and quantification of LD dispersion (**l**) in WT and AMPK^−/−^ MEF cells loaded with OA and unloaded without glucose for 16 h. Cells were fixed and stained with Nile red (LD, red) and DAPI (nucleus, blue). *n*=3. Data are represented as mean±s.e.m. *P<0.05; **P<0.01; ***P<0.001 by *t*-test. Scale bars, 7.5 μm (**b**) and 25 μm (**d**,**k**).

Since during unloading in the absence of glucose cells reorganizes the network of ΔY-MTs, which in turn increases LD dispersion and consumption, we analysed what controlled reorganization of the ΔY-MTs. AMPK is the main sensor of cellular energy status^28^: the increase in the intracellular AMP/ATP ratio caused by energy depletion promotes phosphorylation and activation of AMPK. Activated AMPK drives global cellular changes to adapt cells to nutrient starvation and committed to increase metabolic energy. We confirmed in our system that AMPK and the acetyl coenzyme A carboxylase (ACC, a downstream substrate of AMPK) were phosphorylated after 16 h of the unloading without glucose (Fig. 5a). Interestingly, at 8 h, phosphorylation of AMPK and ACC was significantly reduced in the cells that were initially loaded with LDs when compared with control cells (Supplementary Fig. 1l,m), suggesting that the LDs themselves regulate the cellular energy status. However, at later time points, AMPK remained activated but we did not observe differences in the phosphorylation levels of the kinase in cells with or without LDs, suggesting that additional cellular mechanisms exist to compensate for the AMP/ATP ratio.

AMPK regulates both beta-oxidation^28^ and lipolysis in adipose tissue^29^; however, there has been no evidence relating AMPK to LD location. Thus, we analysed whether AMPK also regulates LD dispersion. Loaded cells were additionally treated for 16 h with loading media (containing glucose and FAs) but in the presence of the AMPK activators, aminoimidazole-4-carboxamide (AICAR) or Metformin^30^,^31^. Interestingly, AMPK activation promoted LD dispersion even in the presence of FAs (Fig. 5b–e). Further, video microscopy analysis demonstrated that AMPK activation highly increased the number of mobile LDs in loaded cells (Fig. 5f and Supplementary Movie 5). Further supporting the role of AMPK in LD mobility and redistribution, inhibition of AMPK during unloading, by means of Compound C^31^,^32^, highly reduced LD mobility, dispersion and consumption (Supplementary Fig. 6a–d and Supplementary Movie 5).

To analyse further whether AMPK is required for LD dispersion and consumption, *wt* MEF and AMPKα-null MEF^33^ (Fig. 5g) were loaded with OA for 24 h. Interestingly, when treated with an identical FA concentration, the AMPKα-null cells accumulated nearly two times more LDs than *wt* cells; clearly suggesting a role of AMPK in LD consumption (Fig. 5h). Then, in order to promote a comparable LD accumulation, *wt* MEFs were loaded with 400 μg ml^−1^ OA and AMPKα-null MEF with 200 μg ml^−1^ OA. After loading, both cell types now showed similar LD levels (Fig. 5i). Next, these cells were unloaded for 16 h in the absence of glucose, and LD consumption and distribution were analysed. Interestingly, the AMPKα-null MEF showed a significantly reduced LD consumption (Fig. 5i) and exhibit a significantly reduced LD dispersion (Fig. 5k,l). Combined, these results support the hypothesis that in the absence of glucose the activation of AMPK increases LD mobility, dispersion and consumption.

The activation of the AMPK regulates autophagy/mitophagy^34^, and thus directly determines mitochondrial and LD dynamics^35^. However, autophagy was not significantly activated in our experimental conditions (Fig. 1i). Thus, we evaluated whether AMPK regulates LD metabolism by inhibition of ACC, an enzyme that increases lipogenesis and reduces beta-oxidation^36^. During nutrient starvation, AMPK phosphorylates and inhibits ACC, thus reducing the conversion of acetyl-CoA to malonyl-CoA. Malonyl-CoA provides two-carbon units for the synthesis of new FAs and simultaneously, like ETO (Fig. 1h), inhibits the carnitine-mediated transfer of FAs into the mitochondria. Thus, in order to test the possibility that AMPK is promoting LD dispersion by inhibition of ACC, we loaded cells with OA but in the presence of 5-Tetradecyloxy-2-furoic acid (TOFA), an allosteric inhibitor of ACC^37^. In contrast to the AMPK activators, AICAR and Metformin (Fig. 5b–e), the TOFA-mediated inhibition of ACC did not modify LD distribution (Supplementary Fig. 6f), suggesting that AMPK promotes LD dispersion by an ACC-independent mechanism. However, in these experiments the TOFA-treated cells showed a reduced accumulation of LDs (Supplementary Fig. 6f). Thus, to test the role of ACC during LD metabolism, cells were now unloaded for 16 h without glucose but in the presence of TOFA. Interestingly, the cells treated with TOFA exhibited a higher LD consumption (Fig. 5j) likely reflecting the described mitochondrial activation occurring after ACC inhibition^36^. These results, in combination with the experiments using ETO (Fig. 1g,h), demonstrate that LD consumption during glucose depletion is tightly regulated by the activity of mitochondria. Therefore, AMPK promotes LD consumption by two independent but simultaneous pathways: (i) AMPK increases LD mobility, dispersion and interaction with mitochondria and (ii) AMPK inhibits ACC to increase the transport of FAs into the mitochondria^36^ and LD consumption.

**Figure 6:**
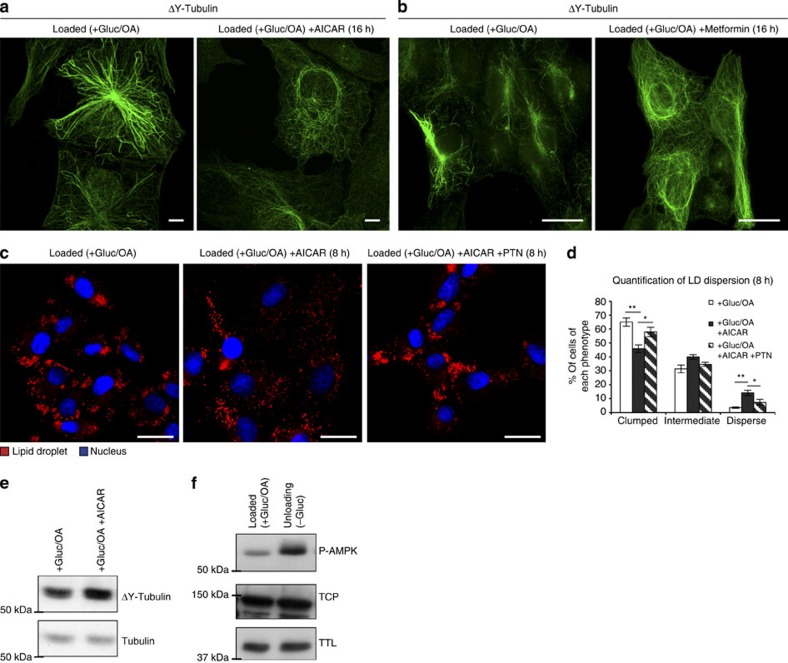
AMPK promotes LD dispersion through MT detyrosination. (**a**) Confocal images of loaded cells treated for 16 h with glucose and FAs in the absence (+Gluc/OA) or presence of AICAR 2.5 mM (+Gluc/OA +AICAR), fixed and labelled with an antibody for detyrosinated tubulin. (**b**) Images of loaded cells treated for 16 h with glucose and FAs in the absence (+Gluc/OA) or presence of Metformin 50 mM (+Gluc/OA +Metformin). Cells were fixed and labelled with antidetyrosinated tubulin antibodies. (**c**,**d**) Images (**c**) and quantification of LD dispersion (**d**) in loaded cells treated with OA for 8 h (+Gluc/OA) or treated simultaneously with 2.5 mM AICAR (+Gluc/OA+AICAR) or 2.5 mM AICAR+15 μM Parthenolide (+Gluc/OA+AICAR +PTN). Cells were fixed and stained with Nile red (lipid droplet, red) and DAPI (nucleus, blue). *n*=5. (**e**) Determination of detyrosinated tubulin and tubulin levels in cells treated as in **a**. (**f**) Western blot analysis of phosphorylated AMPK, TCP, TTL in loaded cells treated for 16 h in a medium with glucose and FAs (+Gluc/OA) or without glucose (−Gluc). Data are represented as mean±s.e.m. *P<0.05; **P<0.01 by *t*-test. Scale bars, 7.5 μm (**a**) and 25 μm (**b**,**c**).

We finally tested the possibility that AMPK increases LD dispersion by affecting the network of MTs. This is a likely mechanism since AMPK has been related to MT dynamics during cell migration^38^ and differentiation of epithelial cells^39^. Thus, we analysed whether the effect of AMPK on LD dispersion might in part be caused by regulation of ΔY-MTs. Interestingly, in addition to promoting LD dispersion, activation of AMPK with AICAR increased the levels of ΔY-tubulin in Vero cells, even in cells loaded for 24 h with FAs and glucose (Fig. 6e). Conversely, inhibition of AMPK with Compound C reduced the levels of ΔY-tubulin in cells unloaded in the absence of glucose for 16 h (Supplementary Fig. 6e). Further, immunofluorescence confirmed that AMPK activation with AICAR or Metformin (Fig. 6a,b) in loaded cells promoted the assembly of the noncentrosomal arrays of ΔY-MTs, characteristic of the cells unloaded without glucose. Finally, suggesting that the effect of activated AMPK on LD dispersion was via ΔY-MTs, after just 8 h, PTN significantly inhibited the AICAR-promoted LD dispersion (Fig. 6c,d). However, the expression of the enzymes involved in ΔY-MT formation (TTL and TCP, Fig. 3e) were not modified during glucose starvation (Fig. 6f), suggesting that alternative and more complex AMPK-regulated mechanisms determine the quality and organization of the MT network.

## Discussion

Here we demonstrate that FAs from LDs are channelled preferentially to different metabolic fates depending on the cellular energetic status: to phospholipids in the presence of glucose or to mitochondrial beta-oxidation in the absence of glucose. When the FAs are removed from the media, cells activate utilization of LDs by increasing LD mobility and dispersion along MTs. In the absence of glucose, this redistribution involves the network of ΔY-MTs and increases the number of LD–mitochondrial contacts, allowing improved FA oxidation. Blocking LD dispersion reduces the number of LD–mitochondrial contacts and impairs beta-oxidation and LD consumption. It is likely that proximity increases the efficiency of the FA exchange between both organelles: in other situations where FA oxidation must be efficient (for example, skeletal muscle after training) the number of interactions LD–mitochondria also increases^40^. These LD–mitochondria contacts can be quite robust, with video microscopy revealing mitochondria that are dragged by LDs, suggesting at least temporary physical docking. Although poorly understood^41^, docking can be mediated by proteins such as perilipin-5 in highly oxidative tissues^42^ and intriguingly by SNAP23 (ref. 43), a protein-bridging membrane during vesicle fusion.

AMPK is the main sensor of cellular energetics^28^ and it was connected to MT dynamics in recent studies^38^,^39^. However, that AMPK regulates post-translational MT modifications and mediates the intracellular transport of organelles was previously unknown. In this sense, we provide the first evidence that AMPK activation reorganizes the ΔY-MTs and highly increases LD mobility to promote the organelle’s re-allocation. Confining LDs and mitochondria to this restricted subset of roads allows increased opportunity for interaction. Thus, we support the hypothesis that the diverse MT tracks, labelled with distinct post-translational modifications, control the specificity of interorganelle contacts^44^. Simultaneously, AMPK-mediated inhibition of ACC increases LD consumption, likely reflecting improved transport of FAs into mitochondria^36^. Further work is now needed to determine how AMPK promotes LD motility, reorganizes the MT network and how the re-organized network of ΔY-MTs drives LD dispersion. Nevertheless, kinesins are the motor proteins proposed to drive LD movements to the cell periphery^45^,^46^,^47^ and some kinesins preferentially binds to ΔY-MTs^48^. Further, the kinesin motors’ landing rates are higher on ΔY-MTs^49^; therefore, a plausible model is that the increase in ΔY-MTs increases the number of motors that are simultaneously active, improving overall LD transport. The results shown here suggest that kinesin-2 is the motor protein that, at least partially, drives LD movement during starvation.

In conclusion, among the exquisite repertory of signal transduction pathways governed by AMPK and committed to produce metabolic energy in response to nutrient scarcity, here we demonstrate a different but complementary mechanism of action. We show that AMPK promotes the reorganization of the MT cytoskeleton to favour the interaction and a dynamic crosstalk between the two key organelles involved in this metabolic adaptation. Many diseases arise from the central role of bioenergetics in cell biology and thus it is not surprising that altered LD accumulation frequently correlates with disease. In this sense, we demonstrate that Metformin and TOFA, currently used for the clinical treatment of prevalent diseases such as diabetes and cancer, dramatically modify LD metabolism. Indeed, further understanding of the coordinated and dynamic relationship existing between two strategic nodes of cellular energetics, LDs and mitochondria, undoubtedly also provides additional insights into these disease conditions.

## Methods

### Reagents, cell culture and treatments

Diethylumbelliferyl phosphate, ETO, 2-deoxyglucose, Compound C, AICAR, 1,1-Dimethylbiguanide hydrochloride (Metformin), PTN and BAF were purchased from Sigma-Aldrich (St Louis, MO, USA). TOFA was purchased from Santa Cruz Biotechnology (Dallas, TX, USA). Nocodazole was purchased from Calbiochem (La Jolla, CA, USA). Nile red, Hoechst-33258 and Deep-Red MitoTracker were from Molecular Probes. Trypsin/EDTA was from Life Technologies. Vero cells (ATCC CCL81), SV40 immortalized AMPK DKO and wild-type (WT) MEFs (a kind gift from Dr Benoit Viollet, INSERM, Paris), COS-1 cells (ATCC CRL-1650) and C2C12 cells (ATCC CRL1772) were cultured in Dulbecco’s modified Eagle’s medium (DMEM, Biological Industries) 10% v/v fetal bovine serum (Biological Industries). Unless specified, the experiments were performed using Vero cells. Media were supplemented with 4 mM L-glutamine, 1 mM pyruvate (Sigma-Aldrich) and with 50 U ml^−l^ Penicillin, 50 μg ml^−1^ Streptomycin and non-essential amino acid (Biological Industries).

OA was conjugated to FA-free bovine serum albumin (BSA, Sigma) at a molar ratio of 6:1 in medium. For LD loading, 10^4^ cells cm^−2^ were plated and treated the same day during 24 h with 175 μg ml^−1^ OA conjugated to albumin. Unless specified, all the loadings are performed following this protocol. For unloading experiments, cells were additionally incubated the indicated times in the standard culture medium described above or medium without glucose (supplemented with L-glutamine, penicillin, streptomycin and non-essential amino acids), and in some cases with the indicated drugs. To accumulate radiolabelled FAs in LDs, cells were treated for 12 h with 70.62 μg ml^−1^ of ^14^C-OA (PerkinElmer NEC-317 50 μCi) conjugated to BSA, followed by additional 12 h of treatment with 175 μg ml^−1^ OA.

Silencer siRNAs against human alpha-tubulin acetyltransferase-1 were obtained from QIAGEN in a FlexiTube format, the siRNA target sequences were as follows: 5′- GAGGCTCATAATGAGGTAGAA -3′, 5′- ACCGCACCAACTGGCAATTGA -3′, 5′- AACCGCCATGTTGTTTATATT -3′, 5′- CCCGCCGACCCGGAACCACAA -3′. Silencer siRNA KAP3 (ref. 27) was obtained from Dharmacon, the sequence was 5′- GUGUCGAGUUAGCUACAAAdTdT -3′. The nonrelated siRNA used was against GFP from Thermo Fisher Scientific or nontargetting siRNA from Thermo Scientific Dharmacon. The siRNA transfections were performed using Lipofectamine RNAiMAX reagent (Invitrogen, Life Technologies). All procedures were performed according to the manufacturer’s instructions.

### Lactate measurement

For lactate measurement, 6 × 10^5^ cells were plated in 60-cm^2^ dishes, and after 24 h treated during 16 h with the specified treatments. Finally, the medium was collected and lactate measure was carried out by the CORE facility of ‘Hospital Clínic i Provincial de Barcelona’ using molecular absorption spectrometry.

### High-resolution respirometry

Overall, 10^6^ cells were collected in DMEM and respiration was measured at 37 °C with high-resolution respirometry using the Oroboros oxygraph. The DatLab software (Oroboros Instruments, Innsbruck, Austria) was used for data analysis. The experimental regime started with routine respiration (*R*), which is defined as basal respiration without additional inhibitors. After observing the steady-state respiratory flux, the ATP synthase was inhibited with Oligomycin (0.5 μg ml^−1^, Sigma) obtaining electron flow coupled to proton pumping to compensate for proton leaks (*L*), followed by uncoupling of oxidative phosphorylation by stepwise titration of FCCP (carbonyl cyanide p-trifluoromethoxyphenylhydrazone, Sigma) up to optimum concentrations in the range of 0.2–2 μM, obtaining maximal respiratory capacity (*E*). Finally, respiration was inhibited by addition of Antimycin A (Sigma) at 2.5 μM (inhibiting complex III) obtaining extramitochondrial residual oxygen consumption (ROX). Respiration ratios were calculated following the manufacturer’s instructions; *R/E* defines the proportion of maximal respiratory capacity used for routine respiration and netR/E is calculated as (*R−L*)/*E* and correspond to the proportion of routine respiration used for ATP production.

### Thin layer chromatography

Cells loaded with radiolabelled FAs were trypsinized, and total lipids were extracted using standard protocols^50^. Briefly, cells were washed with PBS and resuspended in 1 volume of a Methanol:Chloroform (1:2) solution, 1/2 volume of chloroform and 1/2 volume of water were added and tubes were centrifuged. The organic phase was collected, dried, resuspended with chloroform and spotted on 60-Å silica gel TLC plates (Whatmann, Partisil LK6D), and dried for several minutes. A solvent system of chloroform/methanol/water (65:30:5) was added to the TLC chamber and allowed to equilibrate, and plates were run until the solvent front reached the 30% of the plate. Next, after drying, plates were run in a solvent system of hexane/diethyl ether/acetic acid (70:30:1) until the solvent front reached 1 cm from the top of the plate. Radioactive bands corresponding to different lipid species were detected by exposition of a photographic film to the plate by several days. Bands were identified comparing with standards.

### FA beta-oxidation assay

Rates of FA beta-oxidation were determined in cells previously loaded with radiolabelled FAs. Cells were cultured for 16 h at 37 °C in the specified treatment and the released [^14^C]carbon dioxide trapped on filter paper soaked in 1 M potassium hydroxide. The amount of ^14^C radioactivity was determined using a liquid scintillation counter. The rate of beta-oxidation was calculated as the amount of trapped [^14^C]carbon dioxide in relative units produced per mg protein. Results are expressed as the beta-oxidation rate relative to the treatment without glucose.

### Flow cytometry

For the measurement of LD content, cultured cells were trypsinized, washed in PBS and incubated with Nile red solution (5 μg ml^−1^ in PBS) for 15 min at room temperature. To determine neutral lipid staining, samples were subsequently analysed using flow cytometry and yellow emission of Nile red was measured in the 585/42 filter set in a FACSCantoII equipped with a 488-nm Argon laser and a 635-nm red diode laser (Becton & Dickinson, San Jose, CA, USA). To measure mitochondrial membrane potential, after 30-min incubation at 37 °C with 50 nM Tetramethylrhodamine (TMRM, Molecular Probes, Invitrogen), cells were trypsinized, washed in PBS and the TMRM uptake, which reflects the mitochondrial membrane potential (ΔΨm), was determined using flow cytometry in a BD FACS Canto II (BD Bioscience). For assessment of cell death, adhered and detached cells were then pooled, washed and labelled with annexin-V-APC (1:250, BD Pharmigen 550474) and propidium iodide (0.1 μg ml^−l^, Sigma-Aldrich) in a buffer containing 10 mM Hepes (pH 7.4); 140 mM NaCl; 2.5 mM CaCl_2_, for 15 min at room temperature. At least 10,000 cells were analysed in each independent experiment. Samples were subsequently analysed using flow cytometry in a BD FACS Canto II (BD Bioscience). Data from the experiments were analysed using the CellQuest software (Becton & Dickinson). Apoptosis is defined as the percentage of cells positive for annexin V staining and propidium iodide.

### Live cell confocal microscopy

Cells treated as specified were incubated during 10 min at 37 °C with 0.83 μg ml^−1^ Nile red, 3 μg ml^−1^ Hoechst-33258 and 0.3 μM Mitotracker-Deep-red. The cells were examined in a Leica TCS SP5 laser scanning confocal spectral microscope equipped with an incubation control system (37 °C, 5% CO_2_). Images corresponding to single confocal sections were taken using 405-nm laser for Hoechst detection, 514-nm laser for Nile red detection and 633-nm laser for Mitotracker-Deep-red detection, with a × 63 oil immersion objective lens with a numerical aperture of 1.4 and a pinhole of 1.5 a.u. Images were analysed using the Adobe Photoshop CS software (Adobe Systems Inc) and Image J (NIH). Because of the different levels of lipids and membrane potential, the intensity of the dyes varied in the different metabolic conditions used; therefore, in the images shown (but not in the quantifications), the brightness and contrast were adjusted for each image in order to reveal clearly the organelle localization. The proportion of disperse LDs (further than 20 μm to the nucleus) was calculated using the custom-written Matlab code (available on request). Time-lapse video microscopy was performed in the same conditions; images were taken every 0.74 s in 1-min videos and 41.3 s in 2-h videos. Velocity and directionality of LD movement were analysed using the Image J plugin MTrackJ^51^.

### Microscopy analysis of LD dispersion in fixed cells

Cells treated as specified were fixed during 1 h with 4% paraformaldehyde, stained with 4,6-diamidino-2-phenylindole (DAPI) and finally mounted in Mowiol containing Nile red (1:1,000 from a saturated stock solution in acetone). Fluorescence images were taken in a Leica DMI6000 B with a × 63 oil immersion objective lens with a numerical aperture of 1.4. Nile red staining for LDs was evaluated in the Leica filter set L5, while DAPI staining in the Leica filter set A. Images were acquired with a gamma correction of 1.4 to improve Nile red selectivity to LD and reduce background because these images were not used for any intensity quantification. Images were analysed using the Adobe Photoshop CS software (Adobe Systems Inc) and Image J (NIH). LD dispersion was quantified in fixed cells as previously described^21^. Briefly, cells were classified into three classes for LD dispersion: cells with clumped LDs, cells with disperse LDs and cells with an intermediate phenotype. For an example of each type see Supplementary Fig. 1d. Between 100 and 200 cells were analysed per experimental condition in each independent experiment.

### LD analysis in DNA-transfected cells

Cells were plated at 15,000 cells cm^−2^ in glass coverslips. Twenty-four hours after plating, cells were transfected using Lipofectamine LTX (Life Technologies) following the manufacturer’s instructions. The plasmids used were pEGFPC1-LC3 (Addgene, Plasmid 21,073) and alpha-tubulin-GFP (6,117-1, Clontech). pEGFPC1-TTL plasmid was synthesized from TTL cDNA (SC100834, Origene) by standard YUX cloning using primers containing BspEI (forward) and BamHI (backward) restriction sites. Six hours after transfection, cells were treated with 175 μg ml^−1^ OA during 24 h and 16 h in a medium without glucose. GFP-TTL or GFP-LC3-expressing cells were fixed and stained with Nile red. For GFP-TTL experiments, images of epifluorescence microscopy were taken as explained previously. For GFP-LC3 experiments, confocal images were taken with a Leica TCS SP5 laser scanning confocal spectral microscope, using the × 63 oil immersion objective lens. Live cells expressing GFP–tubulin were stained live with Nile red and images were taken each 0.5 s during 1 min. All images were analysed using the Adobe Photoshop CS3 software (Adobe Systems Inc) and Image J (NIH).

### Electron microscopy

Fixation, embedding in Epon and sectioning were performed according to the published techniques^52^. Ultrathin (60 nm) sections were cut on a microtome (model UC6; Leica) and imaged on an electron microscope (model 1,011; JEOL) at 80 kV. Contacts between LDs and mitochondria were quantified in at least six cells per condition (34 cells in total, >700 LDs assessed for mitochondrial association). Association with mitochondria was defined as apposition of a region of the LD surface to the mitochondrial surface as shown in Fig. 2k.

### Immunofluorescence microscopy

For immunofluorescence microscopy, cells grown in coverslips and treated as specified were fixed in cold methanol for 10 min and an additional 15 min in paraformaldehyde 4%; permeabilized in 0.15% Triton X-100 for 10 min; blocked with 1% BSA 0.1% Tween in PBS and incubated for 1 h at room temperature with anti-alpha-tubulin antibody (DM1A Sigma T9026) at 1:250, antidetyrosinated tubulin (Millipore Ab3201) at 1:100, anti-ACSL3 (B01P, Abnova) at 1:200 or anti-ATPsynthase (611450, BD Transduction Laboratories) at 1:1,000 in blocking solution. Finally, primary antibodies were detected with Alexa-conjugated secondary antibodies (Molecular probes) at 1:250 in blocking solution. Confocal images were taken with a Leica TCS SP5 laser scanning confocal spectral microscope, using the × 63 oil immersion objective lens. For 3D-rendering, images were acquired every 80 or 170 nm in the *z* plane for ACSL3 and ATPsynthase images, respectively, and were analysed using the image-processing software Imaris 7.2 (Bitplane), which renders the optical sections into 3D images. Images were analysed using the Adobe Photoshop CS3 software (Adobe Systems Inc) and Image J (NIH).

### Western blot

Cells were washed twice with cold PBS before being scraped into ice-cold 10 mM Tris, pH 7.5, 150 mM NaCl, 5 mM EDTA 0.1% Triton X-100 and a mixture of protease and phosphatase inhibitors. Cells were homogenized by sonication at 4 °C. Protein was quantified with the Bio-Rad Protein Assay kit (Bio-Rad). Western blotting of cells was performed as described previously^53^. Briefly, sample proteins were separated in an acrilamide gel by electrophoresis using Bio-rad Mini-Protean II electrophoresis cell using the manufacturer’s instructions. Then, proteins were transferred from the gel to a polyvinylidene difluoride membrane (Millipore) using the Bio-rad MiniTransBlot electrophoretic transfer cell following the manufacturer’s instructions. After that, membranes were blocked in 5% milk Tween-TBS for 1 h and then incubated overnight with primary antibodies. The primary antibodies used are anti-LC3B (2775, Cell Signaling), anti-alpha-Tubulin (DM1A, Sigma), antidetyrosinated tubulin (AB3201, Millipore), anti-phospho-ACC (3,661, Cell Signaling), anti-AMPK alpha subunit (23A3, Cell Signaling), anti-phospho-AMPK (40H9, Cell Signaling), anti-TTL (14067-1-AP, Protein Tech), anti-TCP (anti-CCP1, 13778-075, Protein Tech), anti-actin (ab40864, Abcam), anti-Acetylated alpha-Tubulin (ab24610, Abcam); all prepared at 1:1,000 in blocking solution. After incubation with primary antibodies, membranes were washed and incubated with peroxidase-conjugated secondary antibodies (1:3,000, Bio-Rad) and detected with ECL (Biological Industries Ltd, Israel) on X-ray films (Fuji Medical). Western blot quantification was performed using the Image J software (NIH). Uncropped scans of all the blots are shown in Supplementary Fig. 7.

### Statistical analysis

All data shown in graphs are the mean and s.e.m., and the statistical significance was determined using the Student’s *t*-test (*P<0.05; **P<0.01; ***P<0.001).

## Additional information

**How to cite this article**: Herms, A. *et al*. AMPK activation promotes lipid droplet dispersion on detyrosinated microtubules to increase mitochondrial fatty acid oxidation. *Nat. Commun.* 6:7176 doi: 10.1038/ncomms8176 (2015).

## Supplementary Material

Supplementary Figures Supplementary Figures 1-7

Supplementary Movie 1 FA depletion promotes MT-dependent LD movement.

Supplementary Movie 2 LD move along microtubules

Supplementary Movie 3 Examples of LD-mitochondrial interactions

Supplementary Movie 4 Detyrosinated-MTs are necessary for LD dispersion

Supplementary Movie 5 AMPK regulates LD movement
