# Supplementary material for: AMPK activation promotes lipid droplet dispersion on detyrosinated microtubules to increase mitochondrial fatty acid oxidation
Source: Nat Commun. 2015 May 27;6:7176. doi: 10.1038/ncomms8176 (PMC4446796; doi:10.1038/ncomms8176)
Supplement: Supplementary Figures — 1-7 [file ncomms8176-s1.pdf]

Supplementary Figure 1. Herms et al.

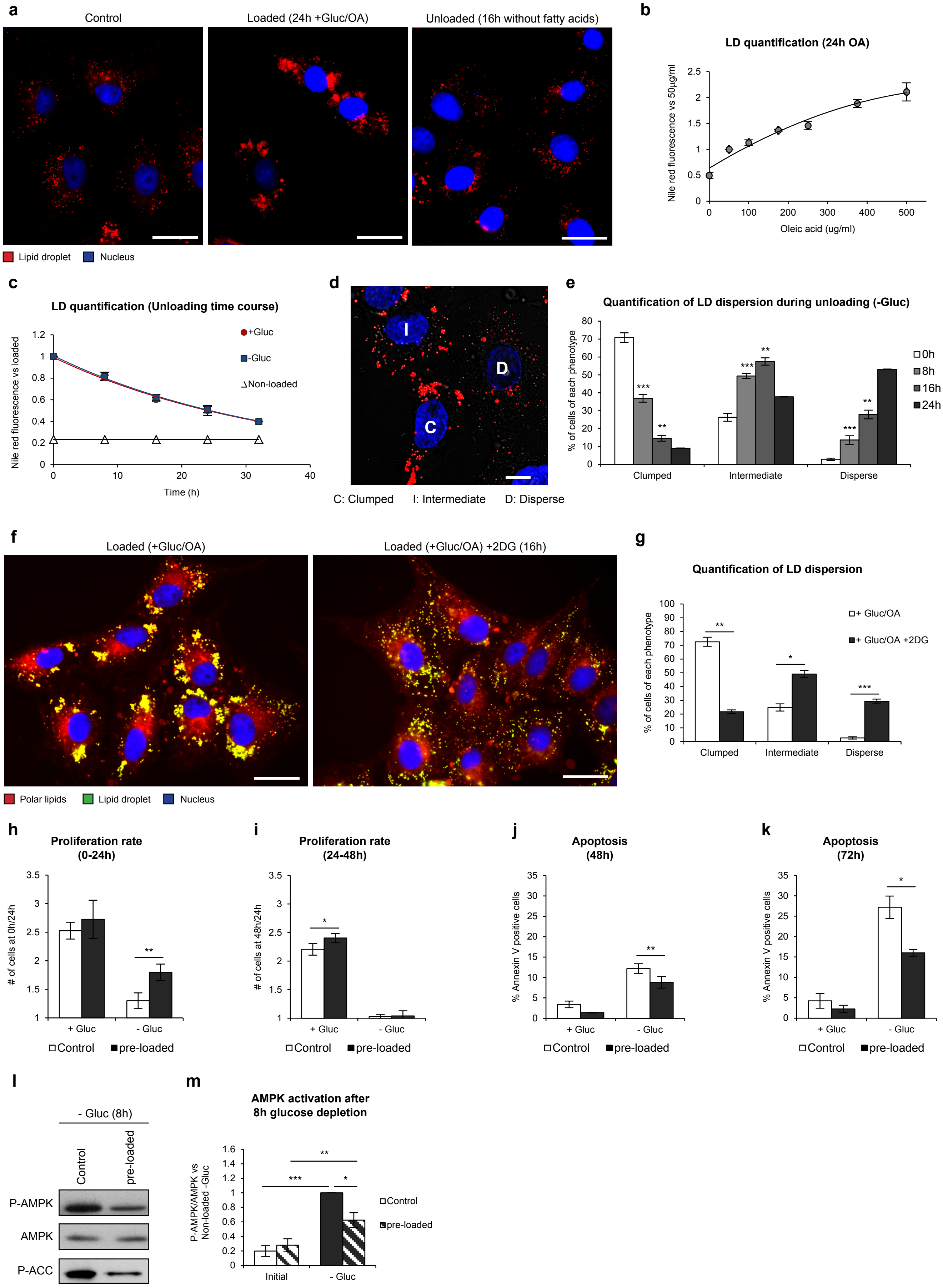

Supplementary Figure 1. LDs metabolism in Vero cells. (**a**) Cells in standard media (left), loaded with oleic acid (middle) or additionally unloaded 16h without added FAs (right). Cells were fixed and stained with Nile red (LD, red) and DAPI (Nucleus, blue). (**b**) LD content in cells treated 24h with increasing oleic acid concentrations. Values expressed with respect to the 50µg/ml treatment. *n*=6. (**c**) LD content of cells loaded with oleic acid and additionally unloaded for different times in the presence (+Gluc) or absence of glucose (-Gluc). *n*=5, 5, 5, 3 and 1 respectively. (**d**) Image of cells stained with Nile red (red) and Hoechst (Nucleus, blue), as an example of the three different phenotypes used to quantify LD dispersion. (**e**) Quantification of LD dispersion loaded cells additionally unloaded for different times in the absence of glucose. *n*=4, 6, 17 and 1 for each time. Statistical significance calculated versus 0h. (**f**, **g**) Images (f) and quantification of LD dispersion (g) in loaded cells treated for 16h with glucose and oleic acid in the absence or presence of 2-deoxyglucose 20µM (2DG). Cells were fixed and stained with Nile red (red for polar lipids and green for neutral lipids) and DAPI. *n* =5. (**h**-**k**) Cells were incubated for 24h in standard media (Control) or loaded with oleic acid (pre-loaded), and additionally treated for the indicated times in a media either containing (+Gluc) or lacking glucose (-Gluc). (h) Ratio between the number of cells after 24h of unloading and the initial number of cells (after loading). *n*=3. (i) Ratio between the number of cells at 48h and the cells at 24h of unloading. *n*=4. (j, k) Percentage of apoptotic cells after 48h (j) and 72h of unloading (k). *n*=3. (**l**) Western-blotting of phosphorylated AMPK (upper panels), total AMPK (middle panels) or phospho-ACC (lower panels) in control and pre-loaded cells treated for 8h without glucose. (**m**) Quantification of the ratio between phosphorylated and total AMPK in cells treated as in (l). *n*=5. Data are represented as mean ± SEM. *P<0.05; **P<0.01; ***P<0.001. by *t*-test. Scale bars, 25µm (a, f) and 10µm (d).

Supplementary Figure 2. Herms et al.

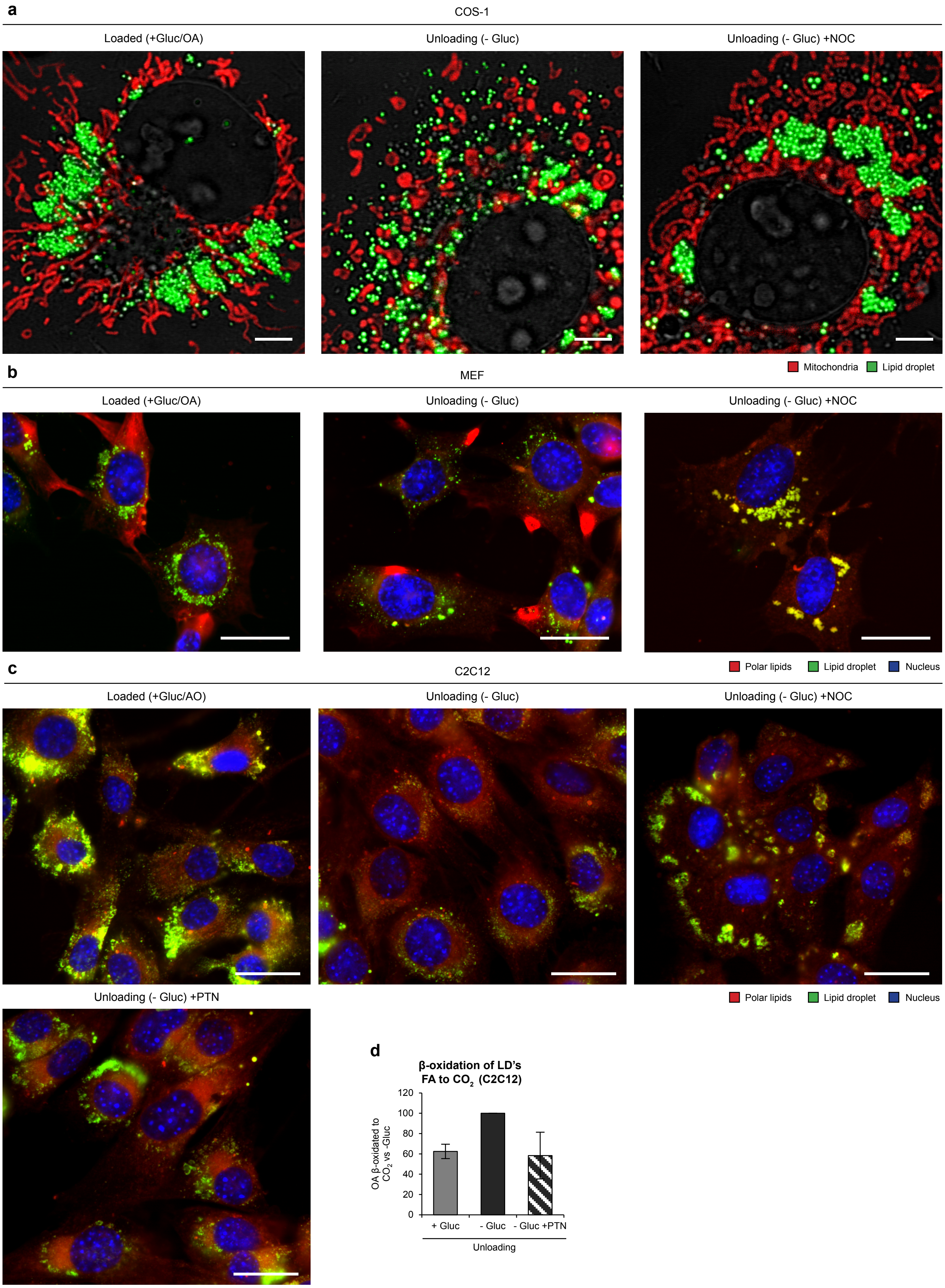

Supplementary Figure 2. LD dispersion in other cell lines. (**a**) Live cell confocal imaging of COS-1 cells loaded and treated for 16h with glucose and FAs (+Gluc/OA), without glucose (-Gluc) or without glucose with Nocodazole 15µM. (**b**) Images of WT MEF cells loaded with oleic acid and additionally treated for 16h with glucose and oleic acid (+Gluc/OA), without glucose (-Gluc), or without glucose but in the presence of 15 µM Nocodazole (-Gluc +NOC). Cells were fixed and stained with Nile red (LD in green and polar lipids in red) and DAPI (Nucleus, blue). (**c**) Images of C2C12 cells loaded with oleic acid and additionally treated for 8h with glucose and oleic acid (+Gluc/OA), without glucose (-Gluc), or without glucose but in the presence of 15µM Nocodazole (-Gluc +NOC) or 15 µM Parthenolide (-Gluc +PTN). Cells were fixed and stained with Nile red (LD in green and polar lipids in red) and DAPI (Nucleus, blue). (**d**) Beta-oxidation in C2C12 cells loaded with radiolabelled FAs in LDs and additionally unloaded for 16h in a media either containing (+Gluc) or lacking glucose (-Gluc) or a media lacking glucose but containing 15µM of Parthenolide (-Gluc +PTN). *n*=3. Data are represented as mean ± SEM. Scale bars, 25µm (b, c), 5µm (a).

Supplementary Figure 3. Herms et al.

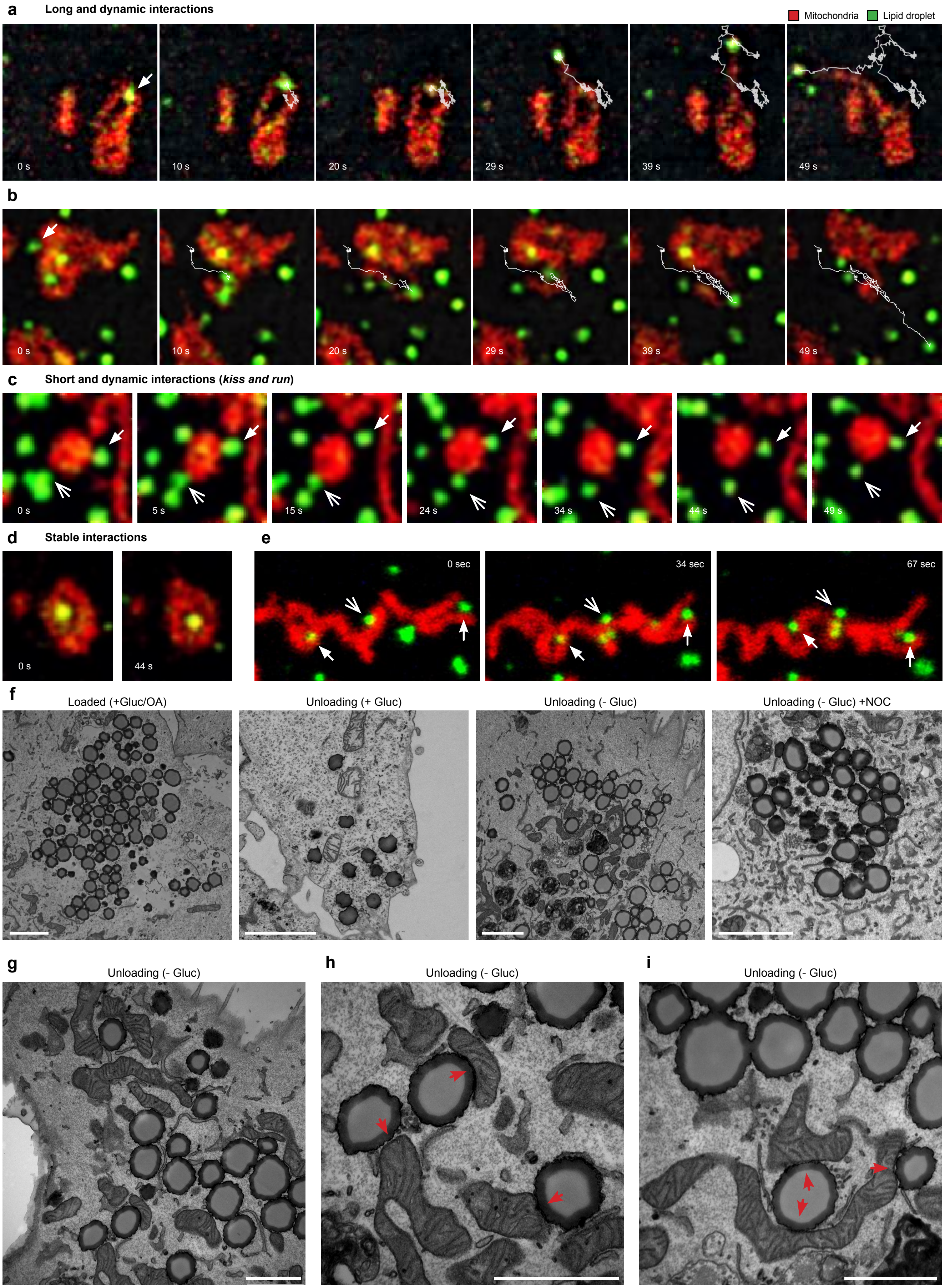

Supplementary Figure 3. Characterization of the LD-mitochondrial interaction. (**a**-**e**) Confocal time lapse video-microscopy of LD-mitochondrial contacts in COS-1 cells loaded 24h with oleic acid and treated 16h without glucose. Cells were stained with Deep-red mitotracker (Mitochondria, red) and Nile red (LD, green) (Video 2). (a, b) Examples of LDs pulling mitochondria. The tracks of the LD movement are highlighted in white. (c) Example of LDs (arrows and open arrows) with kiss and run interactions with mitochondria. (d, e) Examples of long-term interactions between LDs and mitochondria, with LDs completely surrounded by mitochondria (d) and a number of LDs (arrows and open arrow) interacting with a single mitochondria (e). (**f**) Electron microscopy images of Vero cells loaded with oleic acid and additionally treated for 16h with glucose and fatty acids (+Gluc/OA) or unloaded in a media either containing (+Gluc) or lacking glucose without (-Gluc) or with Nocodazole 15µM added. (**g**-**i**) Electron microscopy images showing LD-mitochondria interactions in Vero cells unloaded without glucose. Arrows indicate contacts LD-mitochondria. Scale bars, 2µm (f, g) and 1µm (h, i).

Supplementary Figure 4. Herms et al.

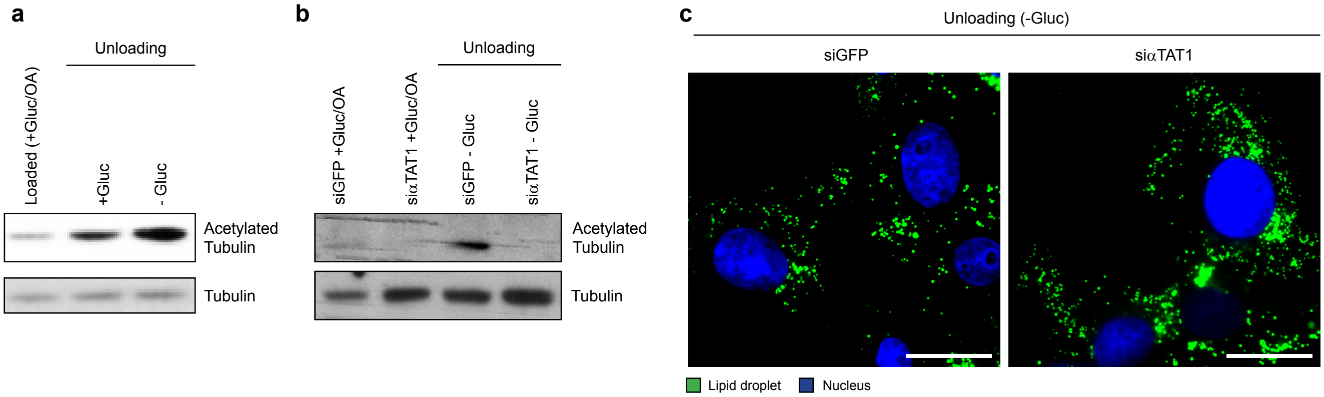

Supplementary Figure 4. Tubulin acetylation is not necessary for LD dispersion. (**a**) Determination of acetylated tubulin and tubulin levels by western blot in loaded cells treated for 16h with glucose and FAs (+Gluc/OA), or unloaded with (+Gluc) or without (-Gluc) glucose. (**b**) Determination of acetylated tubulin and tubulin levels by western blot in cells transfected with an siRNA against GFP as a control or against α-tubulin acetyltransferase (αTAT1), loaded and treated for 16h with glucose and FAs (+Gluc/OA) or without glucose (-Gluc). (**c**) Images of cells transfected as in b, loaded and unloaded for 16h without glucose. Scale bar, 25μm.

Supplementary Figure 5. Herms et al.

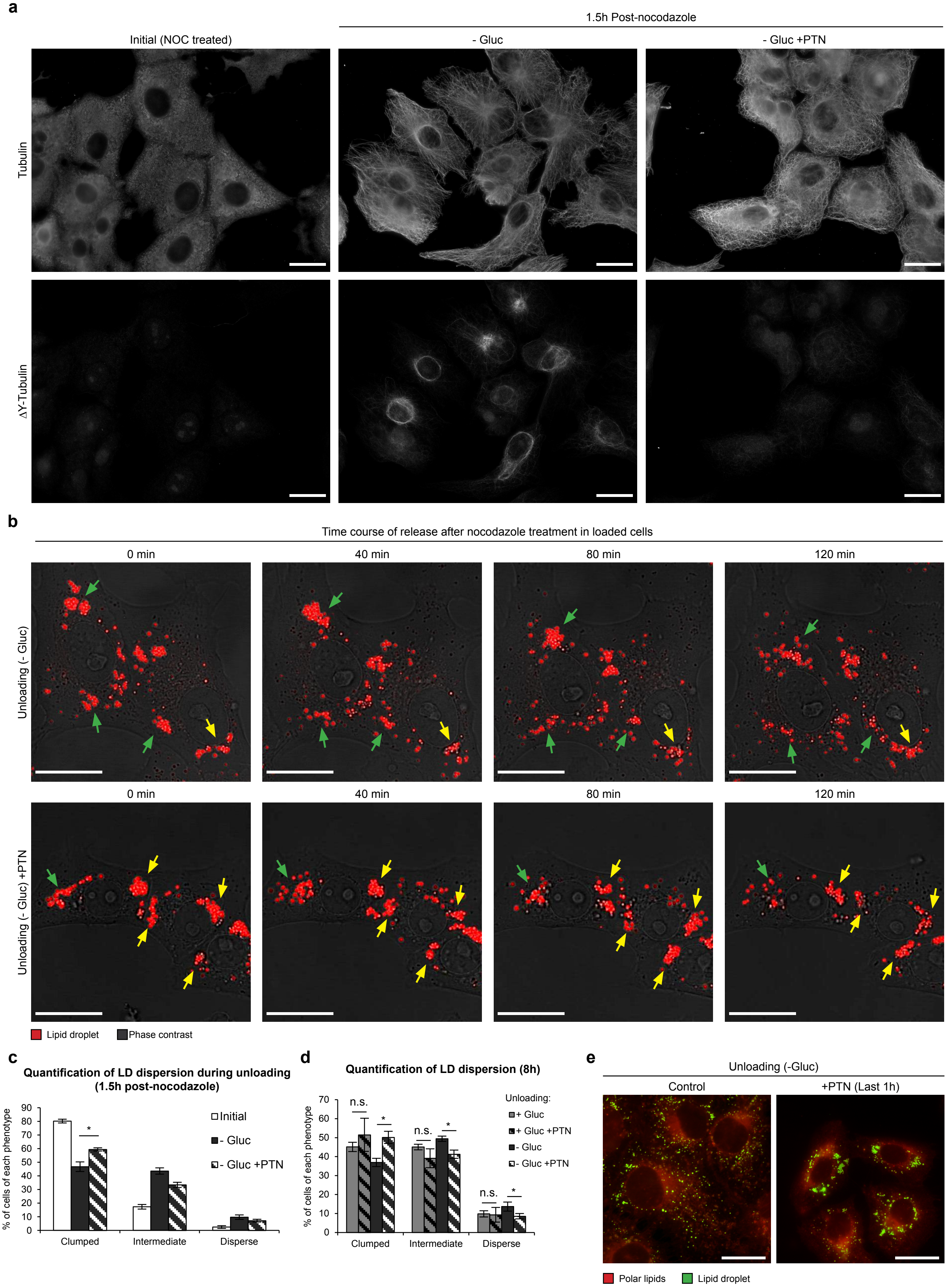

Supplementary Figure 5. Parthenolide (PTN) inhibits MT detyrosination and LD dispersion. (**a**) Cells were loaded with oleic acid and then treated for an additional 4h in a media without glucose and 15µM Nocodazole (Initial). MT organization was analysed with an anti-tubulin antibody (upper panels) or with an anti-detyrosinated tubulin antibody (lower panels). Next, Nocodazole was washed-out and cells were additionally incubated for 1.5h in a medium without glucose in the absence (-Gluc) or the presence of 50µM Parthenolide (-Gluc +PTN). Finally, MT re-organization was re-evaluated with the antibodies. (**b**) Time lapse confocal microscopy of cells treated as in (a) and stained with Nile red (LD, red). In this case, cells were unloaded during 2h in the absence of glucose with or without Parthenolide (Video 4). The phase contrast is shown in grey. Green arrows indicate LD clusters dispersing after just 2h of the wash-out and yellow arrows indicate LDs that remained clumped at the end of the video. (**c**) Quantification of cells with dispersed LDs after the treatment detailed in (b) and a Nocodazole wash-out of only 1.5h in the absence of glucose (-Gluc) or without glucose but with 50µM Parthenolide (-Gluc +PTN). *n*=4. (**d**) Quantification of LD dispersion in loaded cells unloaded for 8h in a media either containing (+Gluc) or lacking glucose (-Gluc); with or without 15µM Parthenolide (PTN). *n*=5-6. (**e**) Cells loaded with oleic acid and unloaded for 16h without glucose (control) or additionally treated the last hour with Parthenolide 50µM. Cells were fixed and stained with Nile red (LD in green and polar lipids in red). Data are represented as mean ± SEM. *P<0.05. by *t*-test. Scale bars, 25 µm.

Supplementary Figure 6. Herms et al.

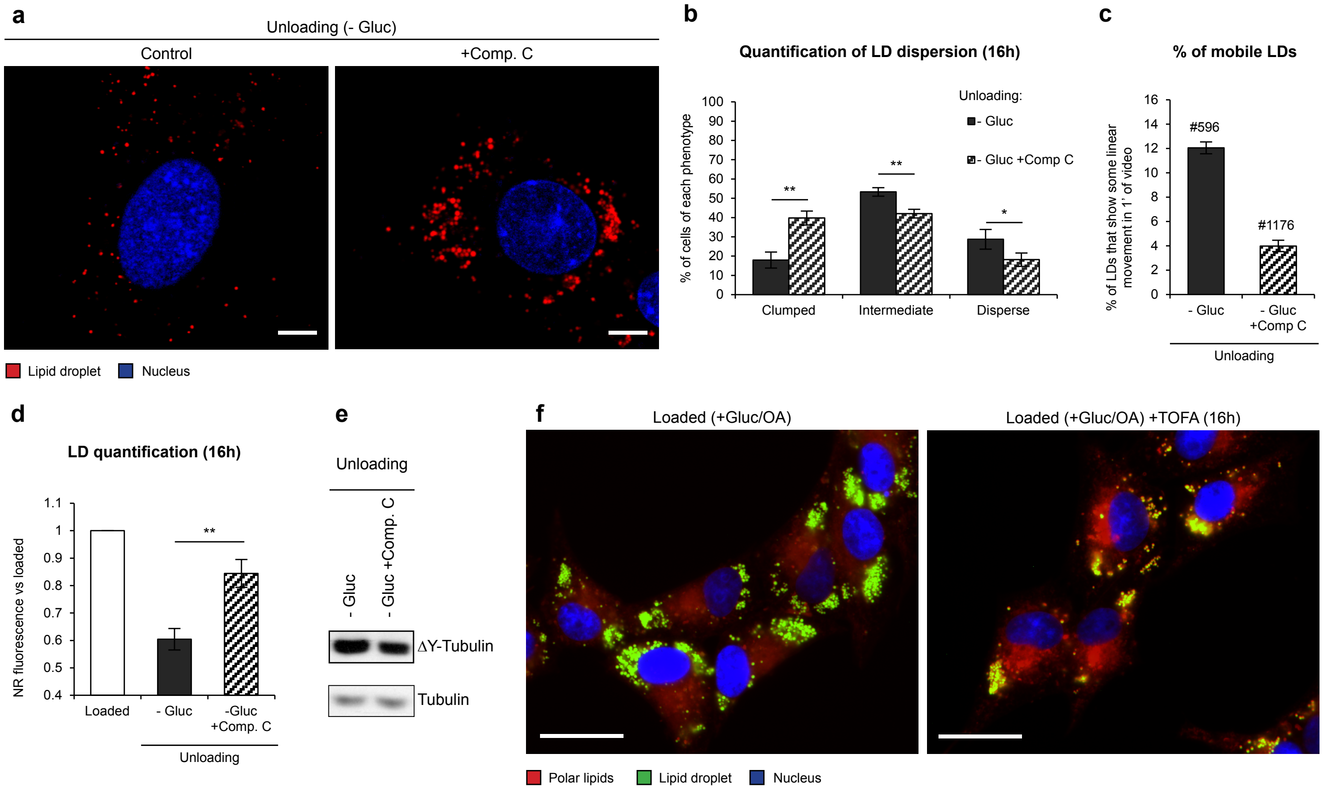

Supplementary Figure 6. AMPK promotes LD mobility and dispersion through MT detyrosination. (**a**) Live cell confocal imaging of cells loaded with oleic acid and additionally unloaded 16h in a media without glucose in the absence (Control) or presence of 10µM Compound C (Comp. C). Cells were stained with Nile red (LD, red) and Hoechst (Nucleus, blue). (**b**) Quantification of LD dispersion in cells treated as in (a). *n*=8. (**c**) Proportion of LDs that show directional movements during 1min in cells treated as in (a) (Video 3). The number of LDs analysed in each condition is specified in the corresponding bar. (**d**) LD content measured by flow cytometry in cells loaded with oleic acid (Loaded) and unloaded 16h without glucose in the absence (-Gluc) or presence of 10µM Compound C (-Gluc + CompC). *n*=7. (**e**) Determination of detyrosinated tubulin and tubulin levels by western blot in cells treated as in (a). (**f**) Cells loaded with oleic acid and treated for additional 16h with glucose and oleic acid in the abscence (+Gluc/OA) or presence of TOFA 30µM. Cells were fixed and stained with Nile red (red for polar lipids and green for neutral lipids) and DAPI (Nucleus, blue). Scale bars, 7.5µm (a) and 25µm (f). Data are represented as mean ± SEM. *P<0.05; **P<0.01 by *t*-test.

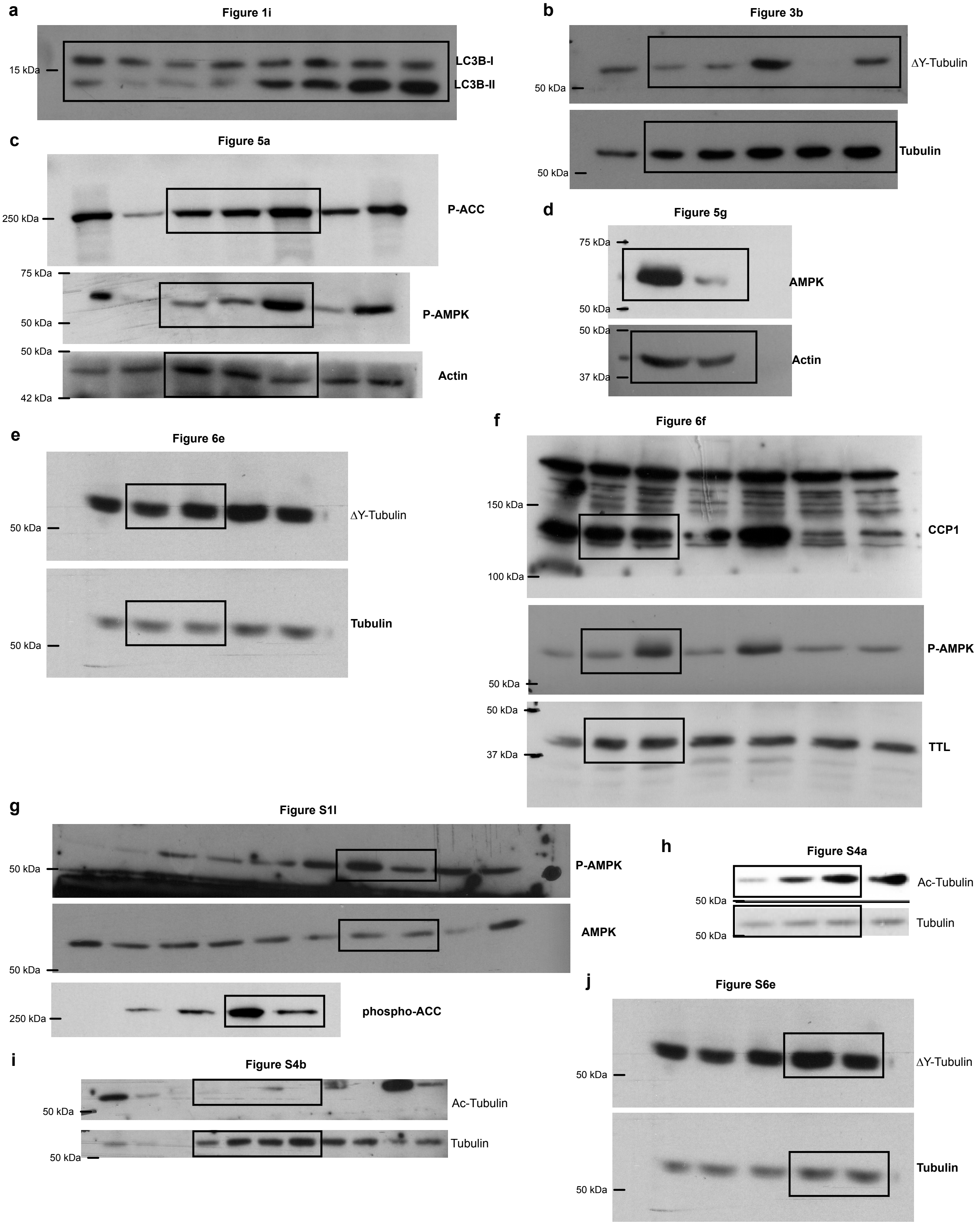

Supplementary Figure 7. Uncropped images of blots. Uncropped scans of blots shown in the Figs 1i, 3b, 5a and g, 6e and f, and supplementary Figs.1l; 4a and b, and 6e.
